# Exaggerated CpH methylation in the autism-affected brain

**DOI:** 10.1186/s13229-017-0119-y

**Published:** 2017-02-17

**Authors:** Shannon E. Ellis, Simone Gupta, Anna Moes, Andrew B. West, Dan E. Arking

**Affiliations:** 1 McKusick-Nathans Institute of Genetic Medicine, Johns Hopkins University School of Medicine, Baltimore, MD 21205 USA; 2 Department of Neurology, University of Alabama at Birmingham, Birmingham, AL 35294 USA

**Keywords:** Autism, Brains, Methylation, Bisulfite sequencing, RRBS

## Abstract

**Background:**

The etiology of autism, a complex, heritable, neurodevelopmental disorder, remains largely unexplained. Given the unexplained risk and recent evidence supporting a role for epigenetic mechanisms in the development of autism, we explored the role of CpG and CpH (H = A, C, or T) methylation within the autism-affected cortical brain tissue.

**Methods:**

Reduced representation bisulfite sequencing (RRBS) was completed, and analysis was carried out in 63 post-mortem cortical brain samples (Brodmann area 19) from 29 autism-affected and 34 control individuals. Analyses to identify single sites that were differentially methylated and to identify any global methylation alterations at either CpG or CpH sites throughout the genome were carried out.

**Results:**

We report that while no individual site or region of methylation was significantly associated with autism after multi-test correction, methylated CpH dinucleotides were markedly enriched in autism-affected brains (~2-fold enrichment at *p* < 0.05 cutoff, *p* = 0.002).

**Conclusions:**

These results further implicate epigenetic alterations in pathobiological mechanisms that underlie autism.

**Electronic supplementary material:**

The online version of this article (doi:10.1186/s13229-017-0119-y) contains supplementary material, which is available to authorized users.

## Background

Autism is a heritable neurodevelopmental disorder affecting one in 68 individuals in the USA [1]. Recent genetic studies have identified a handful of genes that contribute to autism [2], and gene expression studies have begun to unravel how altered gene expression manifests within the autistic brain [3, 4]; however, the majority of risk remains unexplained. In addition to genetic causes, epigenetic mechanisms have been proposed to play an important role in the development of the disorder. Three lines of evidence initially supported this hypothesis. First, direct alterations in epigenetic pathways can dramatically alter early embryonic and neonatal neurodevelopment in the same critical periods as autism-associated changes in the brain [5]. Second, mutations in indirect epigenetic effectors can result in autism-spectrum and related disorders, such as Rett syndrome, Fragile X syndrome, and Angelman syndrome [6]. Finally, deficiencies in DNA methylation (DNAm), historically studied in CpG islands in gene promoters as an indicator of transcriptional repression, have previously been implicated in autism [7–9]. We set out to test for and identify altered methylation within the primary affected tissue in autism—the brain—through analysis of reduced representation bisulfite sequencing data.

## Methods

### Samples

Samples were acquired through the Autism Tissue Program (which has since joined with the Autism Brain Net, https://autismbrainnet.org/). Post-mortem, frozen brain samples from the cerebral cortex Brodmann area (BA) 19 were collected at two different brain banks: the Harvard Brain Tissue Resource Center and the NICHD Brain and Tissue Bank at the University of Maryland with written informed consent having been obtained from next-of-kin or a legal guardian. Work herein was both approved by the IRB of The Johns Hopkins Hospital and University of Alabama at Birmingham and conducted in accordance with institutional guidelines.

### RRBS library preparation

Seventy-one samples were prepared for reduced representation bisulfite sequencing (RRBS). RRBS libraries were prepared using 100 ng of genomic DNA (gDNA). gDNA was first digested with MspI making cuts exclusively at methylated cytosines. 3′ A-overhangs were created and filled in with Klenow Fragments. DNA was then purified using the Qiagen MinElute Kit. Methylated ilAdap PE adapters (Illumina) were ligated to purified gDNA. Fragment size selection (105–185 bp) was carried out by gel extraction on a 2.5% NuSieve GTG agarose gel (Lonza). DNA was purified using Qiaquick Gel extraction Kit eluting DNA in elution bugger pre-warmed to 55 °C. Bisulfite treatment was performed using the ZymoResearch EZ DNA Methylation Gold Kit following the manufacturer’s instructions; however, we eluted with 20 μl M-Elution buffer. Bisulfite-treated DNA was cleaned up using EpiTect spin columns. Samples were PCR amplified (using the following primers: AATGATACGGCGACCACCGAGATCTACACTCTTTCCCTACACGACGCTCTTCCGATC*T and CAAGCAGAAGACGGCATACGAGATCGGTCTCGGCATTCCTGCTGAACCGCTCTTCCGATC*T; * = phosphorothioate bond), and size selection was carried out on a 3% Metaphor agarose gel to ensure that fragments of the correct size (175–275 bp) were amplified. PCR product was cleaned up using the Qiagen minElute column, eluting with elution buffer warmed to 55 °C. Each sample (10 nM) was sequenced in a single lane on the Illumina HiSeq2000 to produce 50 bp single end reads.

### Alignment

Adaptor sequences were removed, and reads shorter than 20 bp were excluded using Trim Galore (v0.2.8). Remaining reads were aligned to hg19 using Bismark (v0.7.7) [10] allowing for one mismatch and setting the seed substring length to 24.

### Methylation estimation

Two separate analyses were carried out based on cytosine context; one for cytosines in the CpG context and a separate analysis for all other cytosines in the genome (CpH, where H = A, C, or T). Samfiles for every sample and each of the two contexts were formatted for input into the R package *methylKit* [11] (v0.9.5) using in-house scripts. Reads were filtered in *methylKit* based on read count discarding bases with coverage below 10× as well as those with coverage greater than the 99.9th percentile of coverage in each sample to remove reads suffering from PCR bias. Data were normalized based on median coverage and methylation percentage estimated using “normalizeCoverage” and “percMethylation,” respectively within *methylKit*.

### Illumina 27K methylation array

To independently verify methylation estimates from RRBS, CpG methylation was also analyzed in 71 cortical brain samples using the HumanMethylation27 BeadChip. These samples comprised 41 controls and 30 autism cases. Data were generated as described previously [12]. Normalized β-values were used for analysis. For comparison to RRBS data, mean methylation was quantified for the 1249 CpGs that directly overlap between the two platforms.

### Sample outlier removal

Four samples were excluded from analysis upon initial diagnostics as their profiles indicated failed library preparation or failed sequencing. In comparison to expected distributions (see Additional file 2: Figure S1a), two were removed due to technical failures, as nearly all (>99%) of their cytosines were methylated after alignment and methylation estimation (see Additional file 2: Figure S1b, c). A third sample was removed because its CpG methylation percentage distribution was not bimodal (see Additional file 2: Figure S1d). The fourth sample was removed because its read coverage distribution did not match the expected distribution (see Additional file 2: Figure S1e).

After identifying samples that failed library preparation and/or sequencing, remaining sample outliers were identified using surrogate variable analysis (SVA) [13], in a manner similar to how outliers have been identified for removal in previous publications [4, 14]. Ten surrogate variables (SVs) were generated using methylation estimates from CpG sites with data across all samples (254,824 CpGs). Samples demonstrating global altered patterns in methylation were identified as any sample that was greater than 4 standard deviations away from the mean in any of the SVs generated. Global outlier samples were removed from analysis. This process was carried out iteratively, and after each round of sample outlier removal, to independently ensure that samples identified as outliers should in fact be removed from analysis; the percentage of known brain meQTLs [15, 16] detected was quantified using a method previously developed for RNA-Sequencing data [14] to guide data analysis. After each round of sample outlier removal, *cis* meQTLs (1 Mb) were detected at SNPs and CpGs present in both the previously reported meQTL studies and the brain data using high-quality genotype data described previously for these samples [14]. meQTLs were detected using *MatrixEQTL* [17] with age, sex, site, and SVs included as covariates, and the percentage of known meQTLs was recorded.

### Single-site differential methylation analysis

To ensure that a single sample’s outlier methylation estimation would not inaccurately inflate the number of sites identified as differentially methylated, methylation outliers at each single site were first identified, as previously described [14]. Briefly, at each tested site, sample outliers were defined as any sample greater than 3 standard deviations away from the mean methylation at that site. These samples were identified at each site and removed from analysis. Further, only variant sites were included for analysis to minimize the multiple testing burden. Accordingly, the 25% least variable sites were excluded from analysis. Single-site differential methylation was then carried out on each site using the *lmFit* function in the *limma* R package [18]. For all cytosines, case-control status was regressed on methylation percentage with age, sex, brain bank, and ten SVs included as covariates (full model). To account for unwanted sources of variation, such as cell type proportion differences or other technical sequencing artifacts, ten SVs were generated using methylation data from all variant sites with data across all samples utilizing the *irw* method from the *sva* package and protecting case-control status. Additionally, as read coverage impacts our confidence in methylation estimates, the log10 of read coverage at each site was included as weights in the model.

Statistical significance was determined by residual bootstrapping, again using *limma*. For each bootstrap, the full model (described above) was fit and residuals recorded. A null model, in which the variable of interest (here, case-control status) was excluded, was also fit. The residuals from the full model were resampled with replacement, randomizing the sample order. “Pseudonull” data were then generating adjusting the fits from the null model with the resampled residuals from the full model. These pseudonull methylation values were then substituted as the outcome variable into the full model, generating a null set of *p* values. These *p* values were collected for each of the 1000 bootstraps to empirically determine study-wide significance.

### Differentially methylated region analysis

Differentially methylated region (DMR) analysis combines nearby sites for analysis and thus benefits from denser methylation data. As such, coverage requirements were relaxed to include sites with at least five reads across 20 cases and 20 controls in both the CpG and CpH data. 1,638,398 CpG and 6,382,340 CpH sites were included for analysis. As above, age, sex, brain bank, and ten SVs were included as covariates. DMRs were then detected using the bumphunterEngine within the R package *bumphunter* (v1.14.0) [19, 20]. Default values were used aside from the following: (1) pickCutoff was set to “TRUE” as to not unnecessarily impose an arbitrary cutoff on the data, (2) the maxgap was set to “300,” in line with previous analyses [19], (3) smoothing was used (TRUE) as it is known that methylation sites tend to be correlated across 300 bp (maxgap), (4) nullmethod was set to “bootstrap” as is required when correcting for covariates, as is the case here, and (5) 1000 bootstraps were carried out (B = 1000). Significance was determined by calculating the family-wise error rate (fwer), the proportion of the 1000 residual boostraps with at least one null candidate region more extreme (defined by having a length longer and higher average value of the regression coefficients) than the region observed. Due to the number of sites included for analysis, chromosomes were analyzed independently, necessitating a multiple test correcting for 24 independent tests (22 autosomes, plus X and Y chromosomes). Significance was determined to be fwer <0.002 (0.05/24).

### Overlap with previous findings

We also tested for altered methylation patterns within the four genomic regions identified as genome-wide differentially methylated in Ladd-Acosta et al. [21] Here, we note specifically that the technology and brain regions are not directly comparable between the studies. This analysis includes RRBS data on 63 individual samples from a single cortical brain region (BA19). Ladd-Acosta et al. studied 41 total samples across three brain regions (temporal cortex, *N* = 16; prefrontal cortex, *N* = 12; cerebellum (*N* = 13) using the Illuina Infinium 450k array, identifying three regions in the temporal cortex samples and one other region in the cerebellar samples to be genome-wide differentially methylated. While one would not expect perfectly overlapping coverage between the data sets given the altered technology, it is possible to query methylation patterns within our samples at the regions reported in Ladd-Acosta et al. To do so, we utilized the DMR data set (at least five reads across 20 cases and 20 controls) to visualize methylation patterns within the regions reported. Methylation patterns were visualized by case-control status, and overlap with significant DMRs was queried.

### Global altered methylation analysis

For each cytosine context, the proportion of sites hypermethylated (defined as mean methylation in cases greater than zero) was calculated at three *p* value cutoffs (0.05, 5 × 10^−3^, and 5 × 10^−4^). To assign significance, this proportion was then compared to the proportion of sites hypermethylated in each of the 1000 residual bootstraps (see Additional file 2: Figure S2). Specifically, to conclude that there was in fact significant global methylation differences between cases and controls at *p* < 0.05, the signal for global alteration differences in the case-control analysis would have to be more extreme than the signal detected in 95% of the residual bootstraps.

### Lists of functional genomic categories

Lists for 28 different functional genomic categories to test for enrichment of hypermethylated cytosines within the CpH context were downloaded from four different sources: (1) the UCSC Genome Browser (mRNA, transcription factor binding sites (tfbs), DNase I hypersensitive sites (dnase), enhancers, CTCF binding sites (CTCF), segmental duplications (segdups), repetitive regions (repeats), and histone marks from lymphoblastoid cell line GM12878 (H3K4m1, H3K4m2, H3K4m3, H3K9Ac, H3K9m3, H3K27Ac, H3K27m3, H3K36m3, H3K79m2, and H4K20m1), (2) UCL Cancer Institute (beacons), (3) the *methylKit* package [11] (promoters, exons, introns, transcription start sites (TSS), CpG islands (CGI), and CGI shores), and (4) the Epigenome Roadmap Project [22] (H327me3.brain, H3K9me3.brain, H3K36me3.brain, H3K4me1.brain, H3K9ac.brain, and H3K4me3.brain) (details in see Additional file 1: Table S1). Brain data from the Epigenome Roadmap project were downloaded from adult cingulate gyrus. For histone marks with data generated on more than one individual (H3K36me3.brain, H3K4me1.brain, H3K4me3.brain, and H3K9me3.brain), the intersection of regions across individuals was utilized for downstream analyses. For beacons, the 200 bp flanking the identified CpG dinucleotide were investigated for enrichment.

### Functional enrichment testing

To test for genomic enrichment of hypermethylated CpH sites in each genomic list and at each *p* value cutoff from the differential methylation analysis (0.05, 5 × 10^−3^, and 5 × 10^−4^), two-sided Fisher’s exact 2 × 2 test was carried out. For each list and at each differential methylation *p* value cutoff, odds ratios and *p* values for enrichment were recorded.

### Power calculation

Power calculations were carried out using the “pwr.t2n.test” function from the *pwr* package in R. This two-sided *t* test of means for samples of different sizes (*N* = 34 controls and 29 cases) was carried out at the 0.05 significance level (Type I error probability).

## Results

To gain a more complete picture of altered DNAm in autism, we carried out Reduced representation bisulfite sequencing (RRBS) in 71 post-mortem cortical brain samples (BA19) at single nucleotide resolution with a quantitative measurement of DNAm across CpG-dense regions of the genome [23]. After sequencing, samples whose libraries failed library preparation, bisulfite conversion, and/or sequencing (*N* = 4) were identified and removed from analysis (see Additional file 2: Figure S1). Further, samples with altered patterns of global methylation patterns, as determined by SVA, were identified and removed from analysis (*N* = 4). While this has been demonstrated to be a sound method for sample outlier removal in RNA-sequencing data previously [14], we ensured that these samples should, in fact, be removed in this RRBS experiment by testing for the proportion of previously identified brain meQTLs detected after the iterative removal of each detected sample outlier. By maximizing the proportion of known meQTLs detected (i.e., true biological signal) (see Additional file 2: Figure S3), this process enabled us to confidently move forward with 63 samples, including 29 autistic cases and 34 controls (see Additional file 1: Table S2).

### Methylation estimation

Methylation was estimated for each sample at cytosines with greater than 10 reads (default in *methylKit* [11]) across at least 20 cases and 20 controls, yielding methylation estimates at 1.0 M CpG and 3.3 M CpH sites (see Additional file 2: Figure S4). In applying these cutoffs, we allow for the inclusion of cytosines with reasonable coverage across a majority of the samples to be included for analysis. On average, samples demonstrated 21.2 and 1.7% methylation across CpG and CpH sites, respectively (see Additional file 2: Figure S5). These values were similar across case-control status with cases having average of 23.4% and controls 19.2% of their CpG sites being methylated. Within the CpH context, cases and controls demonstrated an average of 1.6 and 1.7% percent of cytosines being methylated, respectively (Additional file 2: Figure S5). This is in line with what has been reported previously, where human brain samples have demonstrated 1.3 to 1.5% CpH methylation [24]. To assess the accuracy of the methylation estimates from RRBS, we compared mean methylation percentage estimates at CpGs directly measured by both RRBS and the Illumina 27K array. Given the highly correlated measures of mean methylation (*R* ^2^ = 0.92), we were confident in the methylation estimates acquired through RRBS (see Additional file 2: Figure S6).

### Single-site differential methylation analysis

Individual cytosines were tested for differential methylation regressing case-control status on methylation percentage with age, sex, brain bank, and ten SVs as covariates in both the CpG and CpH context. Statistical significance was determined by residual bootstrapping. No individual CpG or CpH sites were significantly differentially methylated (*p* < 0.05 after adjusting for multiple testing, see Additional File 1: Tables 3-4, see Additional file 2: Figure S7).

### Differentially methylated region analysis

Differentially methylated region analysis was carried out to compare methylation patterns between autism cases and controls in both the CpG and CpH context. This analysis failed to identify any significant DMRs (fwer <0.002) in either the CpG or CpH analyses. The 72 CpG and 54 CpH nonsignificant regions identified by *bumphunter* are included in Additional File 1: Tables 5–6.

### Overlap with previous findings

Within the RRBS CpG data, 56 individual CpGs overlapped with the previously reported differentially methylated regions from Ladd-Acosta et al. [21]. Five CpGs fell within the *PRRT1* region, one was within the *C11orf21* region, 16 within the *ZFP57* region, and 34 were within the *SDHAP3* region. Raw methylation patterns across the three regions containing more than one CpG were visualized (see Additional file 2: Figure S8). While the patterns suggest that there may be differences between autism cases and controls within the *ZFP57* and *PRRT1* regions, it is important to note that raw methylation values have been plotted, which does not account for sources of variation, and secondly, the direction of effect is only consistent with the direction of effect from Ladd-Acosta et al. for the *ZFP57* region (hypermethyion). Conversely, *PRRT1* suggests hypermethylation in the RRBS data; however, it was reported to be a region demonstrating significant hypomethylation previously. Most importantly, however, none of these regions was identified as a significant DMR in the RRBS CpG data.

### Global methylation alterations

**Fig. 1:**
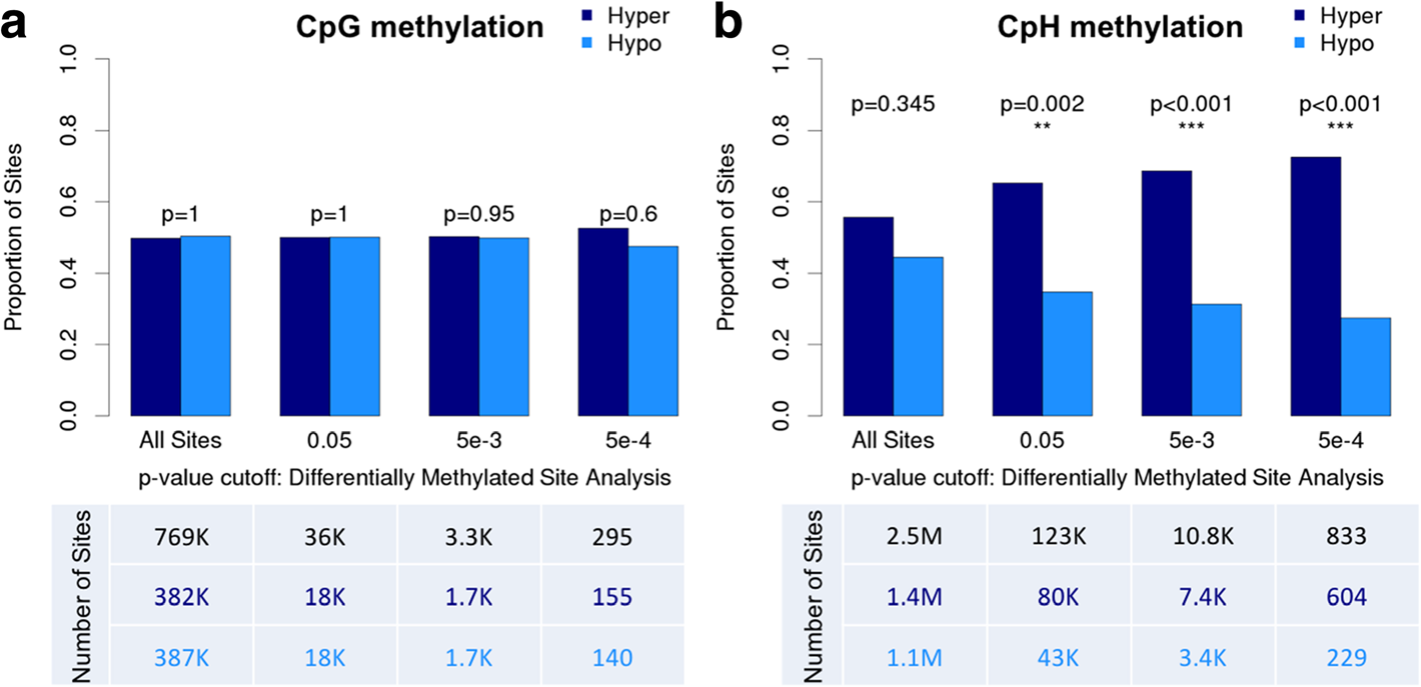
Proportion of hyper- and hypomethylated sites in the CpG and CpH contexts. Proportion of sites (*y-axis*) across increasingly stringent differentially methylated *p* value cutoffs (*x-axis*). The number of cytosines at each differentially methylated *p* value cutoff are displayed in the tables (*below*). **a** With approximately half of all sites demonstrating increased methylation (*navy*) and the other half decreased methylation (*light blue*), CpG sites behave as expected under the null. This pattern holds across increasingly stringent differential methylation *p* value cutoffs demonstrating no global differences in methylation within the CpG context. **b** The proportion of cytosines demonstrating hypermethylation is not significantly different from the proportion demonstrating hypomethylation when looking at all CpH sites; however, with increasingly stringent differentially methylated *p* value cutoffs, there is a significant proportion of hypermethylated CpH sites in the autistic brain

In addition to testing for differential methylation at individual sites, we measured global changes associated with hypo- or hypermethylation. Among sites demonstrating nominal differential methylation (*p* < 0.05), there is a consistent and statistically significant proportion of cytosines demonstrating increased methylation within the CpH context (Fig. 1b, *p* = 0.002 with 65.2% of sites demonstrating hypermethylation), but not the CpG context (Fig. 1a).

Further, given that more stringent *p* value cutoffs for differentially methylated sites should enrich for true positives, we hypothesized that the global hypermethylation signal would increase in strength with increasingly stringent *p* value cutoffs in the CpH analyses, but not in the CpG analyses, which did not yield global differences. Indeed, as more stringent differential methylation *p* value cutoffs were imposed, a greater skewing in the number of hypermethylated to hypomethylated sites was observed (Fig. 1b). As expected, this trend was not seen in the CpG sites (Fig. 1a). Moreover, the effect size of this hypermethylation signal increased with larger methylation differences between cases and controls (see Additional file 2: Figure S9). Taken together, these data suggest that small increases (CpH sites with a differentially methylated *p* value <0.001 demonstrate a median 1.8% increase in cases relative to controls) in methylation across many individual sites are found at cytosines outside of the classically studied CpG context in the autistic brain.

### Functional analysis of hypermethylated CpHs

**Fig. 2:**
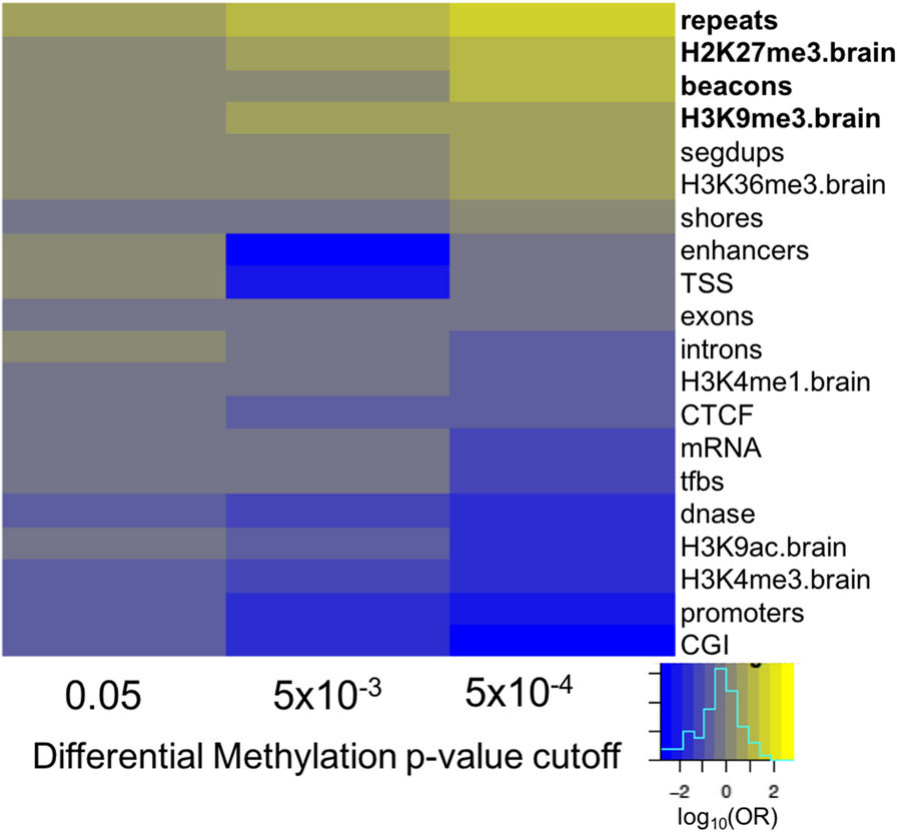
Genomic enrichment of hypermethylated CpH sites. For each genomic category, effect of enrichment (*log odds ratio*) is plotted across increasingly stringent differential methylation analysis *p* value cutoffs (*x-axis*). Enrichment within a genomic category is indicated with the color *yellow*. Categories demonstrating significant enrichment (*p* < 0.05) are in *bold*

To gain insight into how altered CpH methylation (mCH) may be linked to the pathobiology of autism and aberrant neurodevelopment, we tested for enrichment of hypermethylated CpHs in various functional categories annotated across the genome. We used Fisher’s exact test to detect enrichment of hypermethylated cytosines in 20 functional categories of the genome at several thresholds produced in the differential methylation analysis. This analysis highlights a role for increased methylation at CpH sites within repetitive regions of the genome (OR = 1.39, *p* = 5.7 × 10^−4^), in regions that contain non-polymorphic human-specific CpGs, termed beacons [25] (OR = 1.27, *p* = 0.04), and at deactivating histone marks in the brain (H3K27me3: OR = 1.22, *p* = 6.8 × 10^−3^; H3K9me3, OR = 1.22, 1.6 × 10^−2^) (Fig. 2). Of note, histone-specific enrichment was not seen in any of the ten histone marks tested using data generated from a lymphoblastoid cell line, suggesting that this enrichment is tissue-dependent (see Additional file 2: Figure S10).

## Discussion

Previous studies of methylation in autism were limited by the number of sites investigated, a lack of dynamic range in microarrays, the number of samples available for study, and the use of DNA that was procured from cell lines and tissue other than the brain. RRBS, in addition to querying methylation at more sites than the previously used Infinium HumanMethylation450 array (Illumina) [21, 26], enables measurement of methylation at cytosines outside of the classically studied CpG context to include cytosines within the CpH context. mCH is rare in most tissues; however, it accumulates in DNA in human and mouse brain postnatally, ultimately reaching levels similar to that of CpG methylation (mCG) in brain DNA [24, 27, 28]. In contrast to mCG, which remains largely unchanged during postnatal development, mCH accumulation correlates with synaptogenesis and increases especially during the first few years of life [24, 27], a time period of particular interest in autism. Thus, we used post-mortem cortical brain samples to characterize CpG and CpH methylation in autism-affected brain tissue and compared this to matched neurologically normal control brain tissue.

While we did not detect any significant differences in any single CpG or CpH site or region, we report, for the first time to our knowledge, an increase in global CpH methylation within the brains of autism-affected individuals. These findings are enriched within three general functional categories: (1) repetitive regions of the genome, (2) regions that contain beacons [25], and (3) regions of the genome that harbor deactivating histone sites in the brain (Fig. 2). These results are particularly intriguing, as autism is a disorder that includes deficits in language, a key trait unique to humans. Specifically, repetitive regions, defined as regions that contain interspersed repeats and regions of low DNA complexity, account for a substantial amount of variation between humans and other species. Similarly, beacons are regions known to harbor human-specific CpGs, regions of substantial regulatory differences between humans and primates with CpG density at beacons resulting in decreased methylation over time within associated CGIs [25]. Due to the importance of regulation within the human brain, altered regulation of methylation at regions harboring beacons is an important avenue of study within autism. Further, the finding here that increased mCpH occurs in beacons within the autistic brain offers support that beacons may, as a result of human evolution, highlight regions of the genome with particular susceptibilities for human, and particularly neurological [25], disease. Finally, we report enrichment at two deactivating histone marks, H3K27me3 and H3K9me3. Methylation at these histone marks has been reported to decrease accessibility of the surrounding DNA to the transcriptional machinery, resulting in decreased levels of expression [29, 30]. Given previous reports of altered gene expression at transcriptional regulators [31], the finding of altered CpH methylation at deactivating histone marks not only corroborates previous findings but also further suggests a role for general transcriptional suppression at the level of mCH within the autistic brain. This finding offers another possible avenue for study of the role for epigenetic alterations and their effects on transcription in autism. Taken together, this finding implicates increased methylation within autism brain tissue at cytosines outside of the canonical CpG dinucleotide.

It is not clear whether increased CpH methylation in autism is causal, protective, or benign in the etiology of disease. Given that mCH is specifically enriched in both the human and mouse brain [24], future studies can begin to probe the function of CpH methylation in successful and aberrant neurodevelopment.

Several limitations should be noted. To maximize the number of samples that could be sequenced, this study employed RRBS rather than whole genome bisulfite sequencing (WGBS). As RRBS enriches for CpG rich regions of the genome, we are unable to estimate methylation for cytosines outside of CpG rich regions. As sequencing costs continue to decline, WGBS of all the available brain tissue specimens will become more feasible and will undoubtedly add further insight into the role of methylation and other epigenetic phenomenon in autism. Additionally, given the scarcity of samples, sample size is always a cause for concern in post-mortem brain studies. Here, we report findings from the largest number of samples studied to date. As such, we are 80% powered to detect mean methylation differences greater than or equal to 2.6% (see Additional file 2: Figure S11); however, group differences of smaller effect or idiosyncratic changes could have been missed in these analyses. Finally, mCpG can be converted to 5-hydroxymethylcytosine (5hmC) and has been detected within the human brain [32, 33]. As RRBS does not distinguish 5hmC from mCpG, testing for altered methylation within 5hmC specifically may detect differences within the autism brain; however, that question is not answerable with the data generated in these studies.

## Conclusions

We report that while there we did not detect any single CpGs or CpHs that were significantly differentially methylated in autism cases relative to controls, we do report that increased CpH methylation occurs throughout the genome in DNA from autism-affected brain. These CpH sites are strongly associated with repetitive regions, deactivating histone marks, and beacons, offering new insights into how the epigenome may be affected in autism.

## Additional files

Additional file 1: Table S1. Functional genomic region information. **TableS2.** Sample information. **Table S3.** Differentially methylated CpG sites (*p* < 0.05). **Table S4.** Differentially methylated CpH sites (*p* < 0.05). **Table S5.** Differentially methylated CpG regions. **Table S6.** Differentially methylated CpH regions. (XLSX 10,803 kb)
Additional file 2: Figure S1. Detecting sample outliers. **Figure S2.** Null distributions. **Figure S3.** Published meQTLs help identify sample outliers. **Figure S4.** Cytosine summary. **Figure S5.** Percent methylation. **Figure S6.** Correlation between RRBS and 27K array data. **Figure S7.** Single-site differential methylation analysis. **Figure S8.** Methylation patterns at previously reported DMRs. **Figure S9.** Hypermethylation signal in CpH sites increases with methylation difference between cases and controls. **Figure S10.** Functional enrichment testing at histone marks from a lympoblastoid cell line (LCL). **Figure S11.** Power calculation curve. (PPTX 1789 kb)

## Abbreviations

BA: Brodmann area
CGI: CpG island
DNAm: DNA methylation
dnase: DNase I hypersensitive sites
mCG: CpG methylation
mCH: CpH methylation
RRBS: Reduced representation bisulfite sequencing
segdups: Segmental duplications
tfbs: Transcription factor binding site
TSS: Transcription start site
WGBS: Whole genome bisulfite sequencing
