# Supplementary material for: Exaggerated CpH methylation in the autism-affected brain
Source: Mol Autism. 2017 Feb 17;8:6. doi: 10.1186/s13229-017-0119-y (PMC5351204; doi:10.1186/s13229-017-0119-y)
Supplement: Additional file 2: Figure S1. — Detecting sample outliers. Figure S2. Null distributions. Figure S3. Published meQTLs help identify sample outliers. Figure S4. Cytosine summary. Figure S5. Percent methylation. Figure S6. Correlation between RRBS and 27K array data. Figure S7. Single-site differential methylation analysis. Figure S8. Methylation patterns at previously reported DMRs. Figure S9. Hypermethylation signal in CpH sites increases with methylation difference between cases and controls. Figure S10. Functional enrichment testing at histone marks from a lympoblastoid cell line (LCL). Figure S11. Power calculation curve. (PPTX 1789 kb) [file 13229_2017_119_MOESM2_ESM.pptx]

## Slide 1
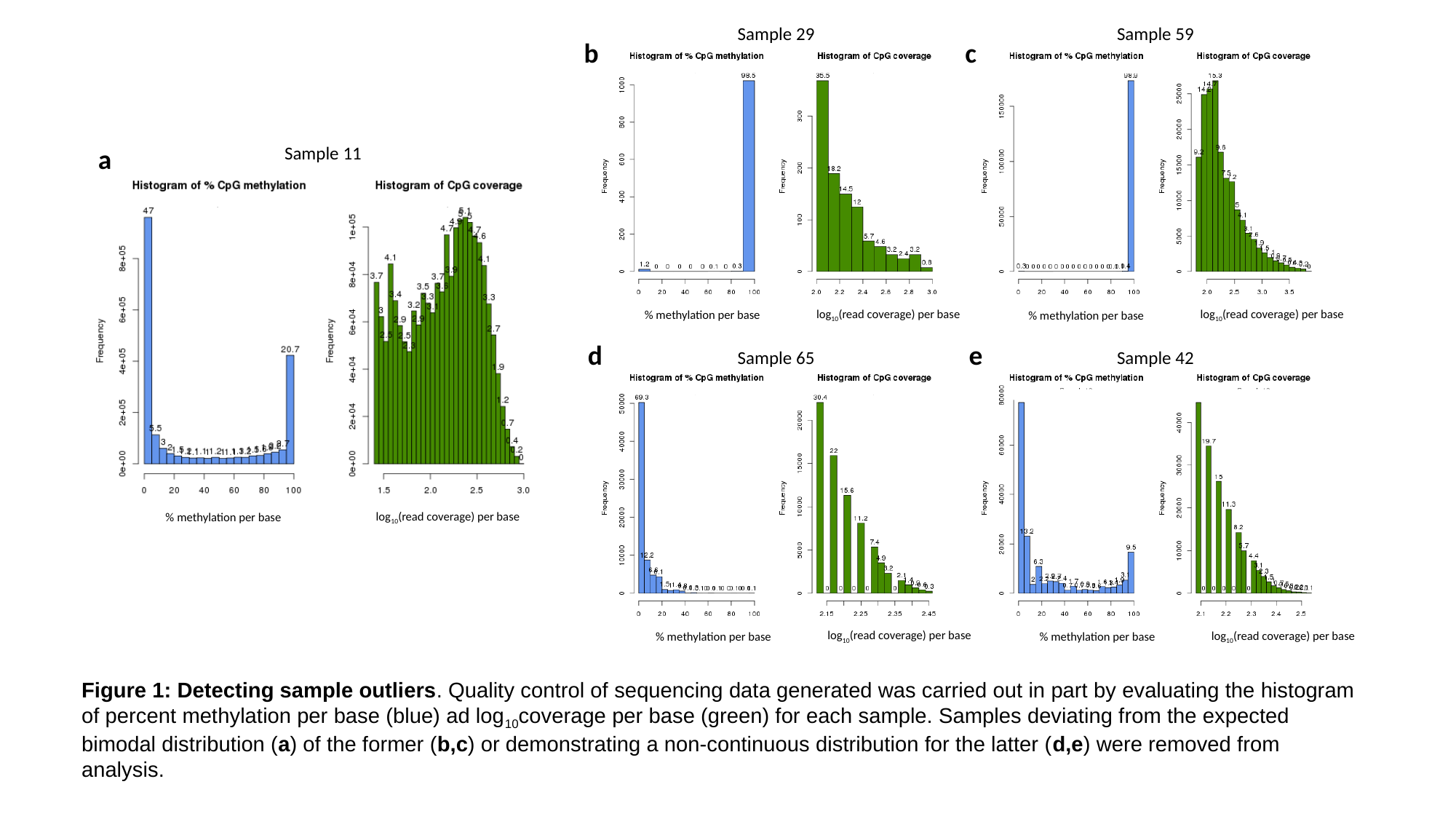

Sample 29
Sample 59
b
c
a
Sample 11
% methylation per base
log10(read coverage) per base
% methylation per base
log10(read coverage) per base
d
e
Sample 65
Sample 42
% methylation per base
log10(read coverage) per base
% methylation per base
log10(read coverage) per base
% methylation per base
log10(read coverage) per base
Figure 1: Detecting sample outliers. Quality control of sequencing data generated was carried out in part by evaluating the histogram of percent methylation per base (blue) ad log10coverage per base (green) for each sample. Samples deviating from the expected bimodal distribution (a) of the former (b,c) or demonstrating a non-continuous distribution for the latter (d,e) were removed from analysis.

## Slide 2
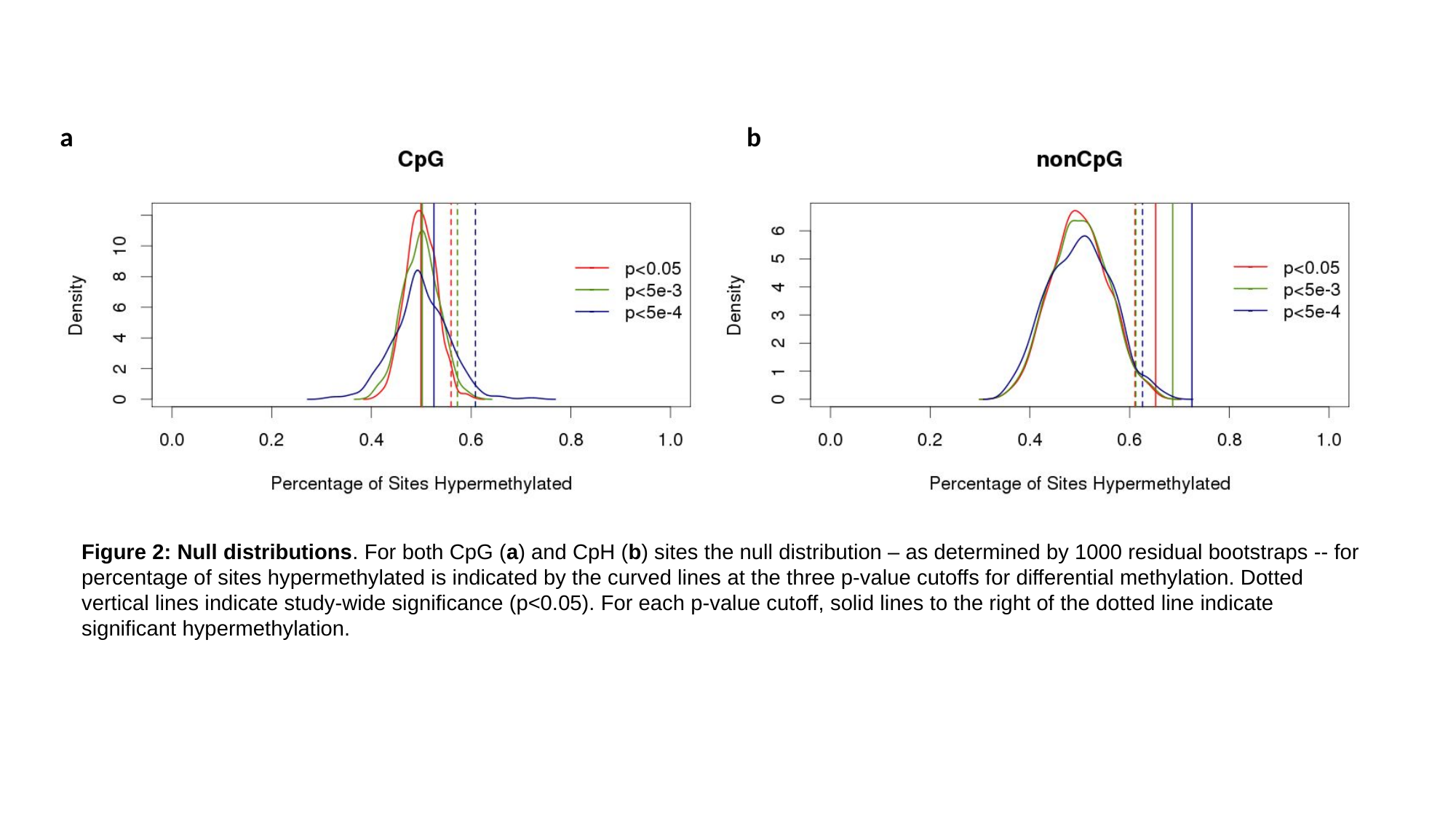

a
b
Figure 2: Null distributions. For both CpG (a) and CpH (b) sites the null distribution – as determined by 1000 residual bootstraps -- for percentage of sites hypermethylated is indicated by the curved lines at the three p-value cutoffs for differential methylation. Dotted vertical lines indicate study-wide significance (p<0.05). For each p-value cutoff, solid lines to the right of the dotted line indicate significant hypermethylation.

## Slide 3
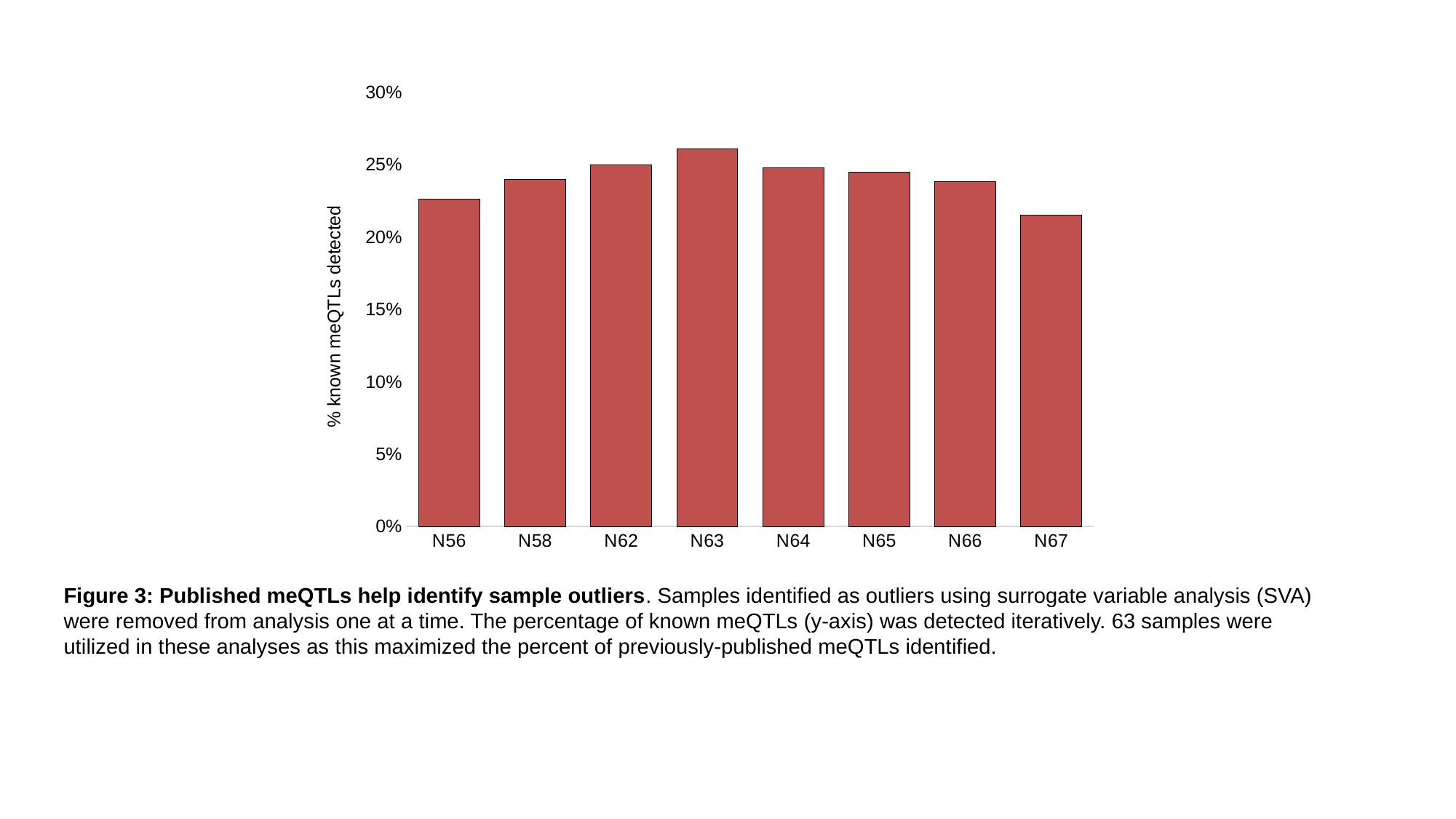

### Chart
| Category | |
|---|---|
| N56 | 0.226480836236934 |
| N58 | 0.240121580547112 |
| N62 | 0.25 |
| N63 | 0.261261261261261 |
| N64 | 0.247761194029851 |
| N65 | 0.244776119402985 |
| N66 | 0.238372093023256 |
| N67 | 0.215116279069767 |Figure 3: Published meQTLs help identify sample outliers. Samples identified as outliers using surrogate variable analysis (SVA) were removed from analysis one at a time. The percentage of known meQTLs (y-axis) was detected iteratively. 63 samples were utilized in these analyses as this maximized the percent of previously-published meQTLs identified.

## Slide 4
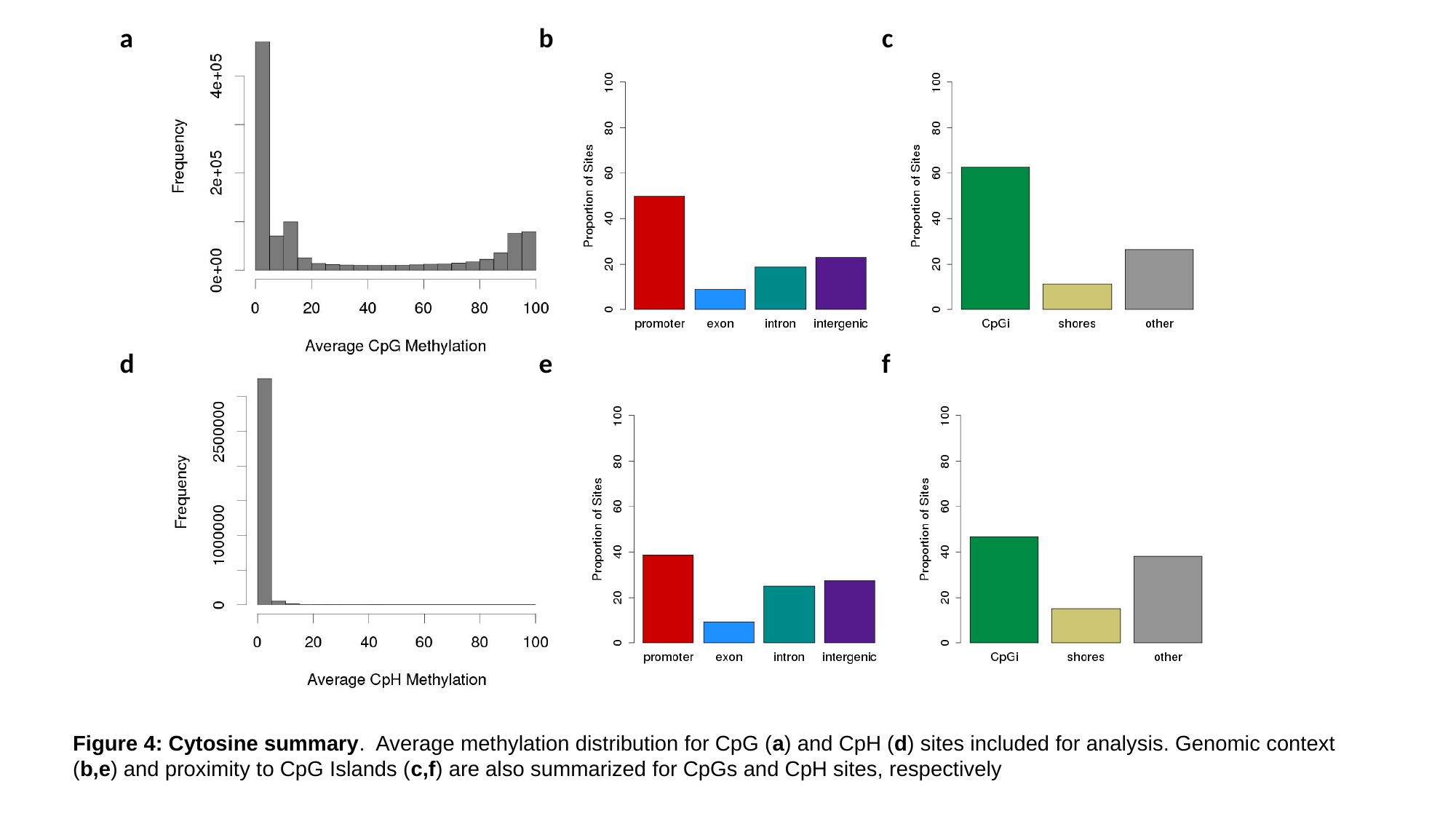

a
b
c
d
e
f
Figure 4: Cytosine summary. Average methylation distribution for CpG (a) and CpH (d) sites included for analysis. Genomic context (b,e) and proximity to CpG Islands (c,f) are also summarized for CpGs and CpH sites, respectively

## Slide 5
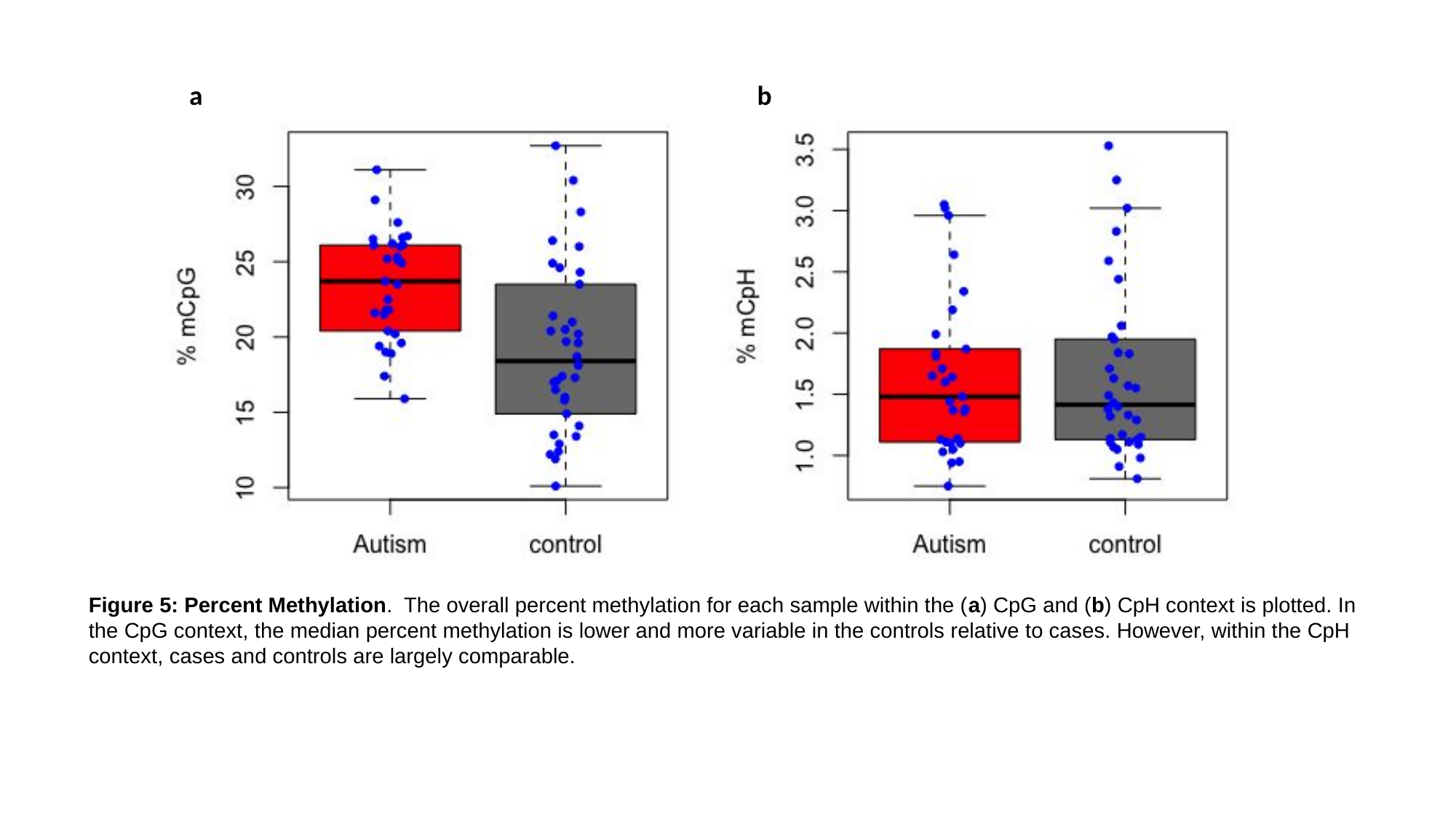

a
b
Figure 5: Percent Methylation. The overall percent methylation for each sample within the (a) CpG and (b) CpH context is plotted. In the CpG context, the median percent methylation is lower and more variable in the controls relative to cases. However, within the CpH context, cases and controls are largely comparable.

## Slide 6
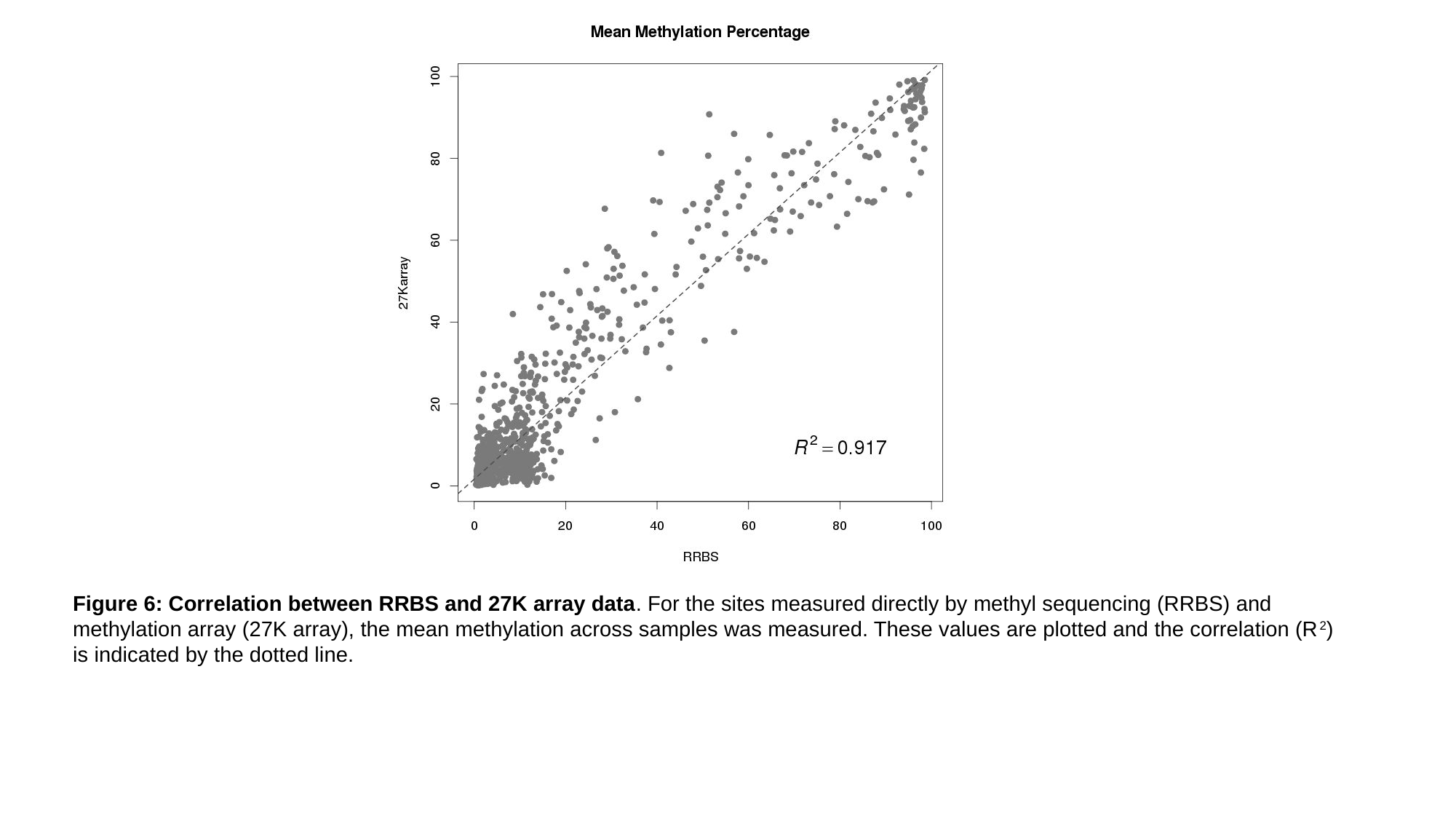

Figure 6: Correlation between RRBS and 27K array data. For the sites measured directly by methyl sequencing (RRBS) and methylation array (27K array), the mean methylation across samples was measured. These values are plotted and the correlation (R2) is indicated by the dotted line.

## Slide 7
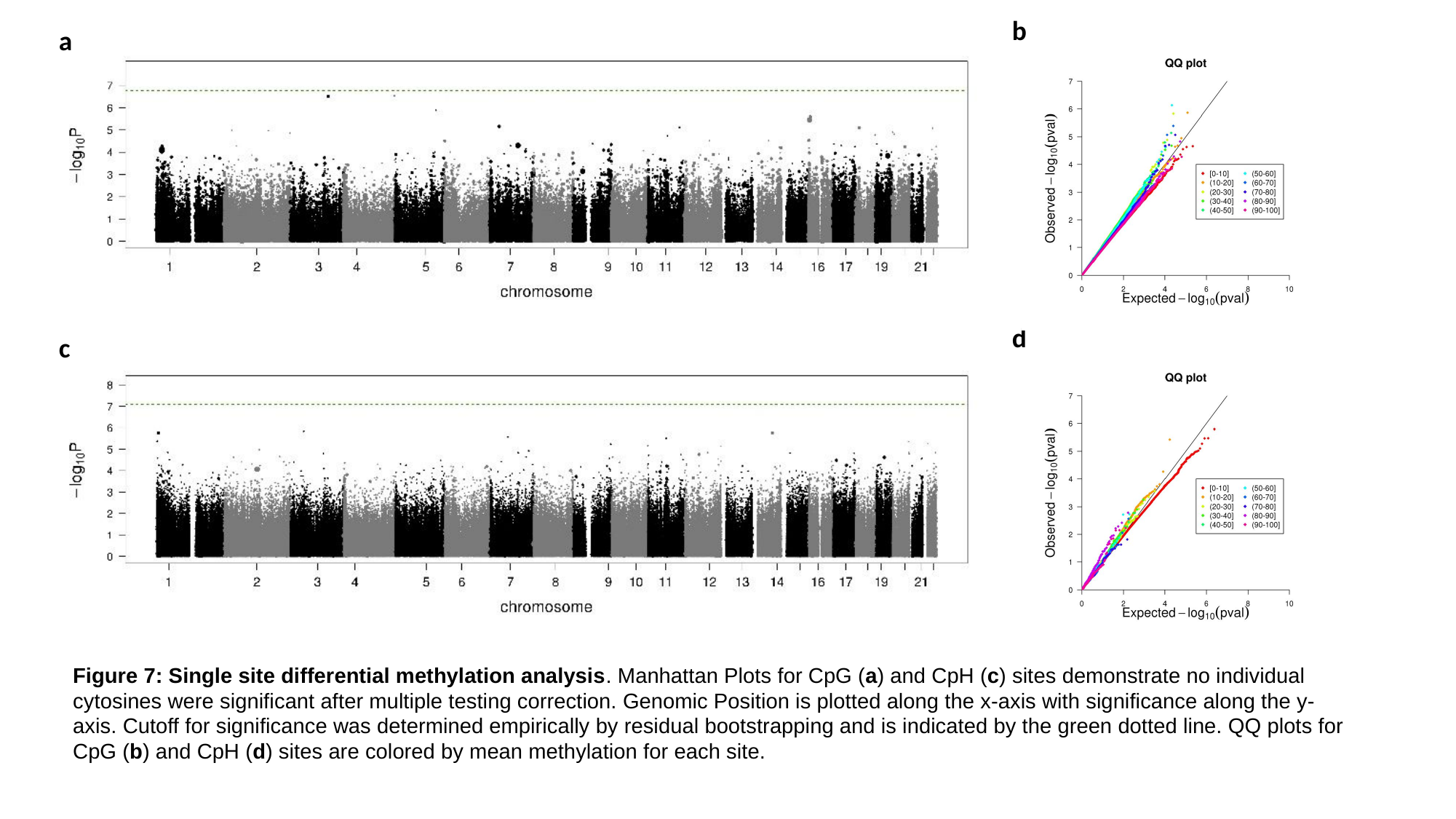

b
a
d
c
Figure 7: Single site differential methylation analysis. Manhattan Plots for CpG (a) and CpH (c) sites demonstrate no individual cytosines were significant after multiple testing correction. Genomic Position is plotted along the x-axis with significance along the y-axis. Cutoff for significance was determined empirically by residual bootstrapping and is indicated by the green dotted line. QQ plots for CpG (b) and CpH (d) sites are colored by mean methylation for each site.

## Slide 8
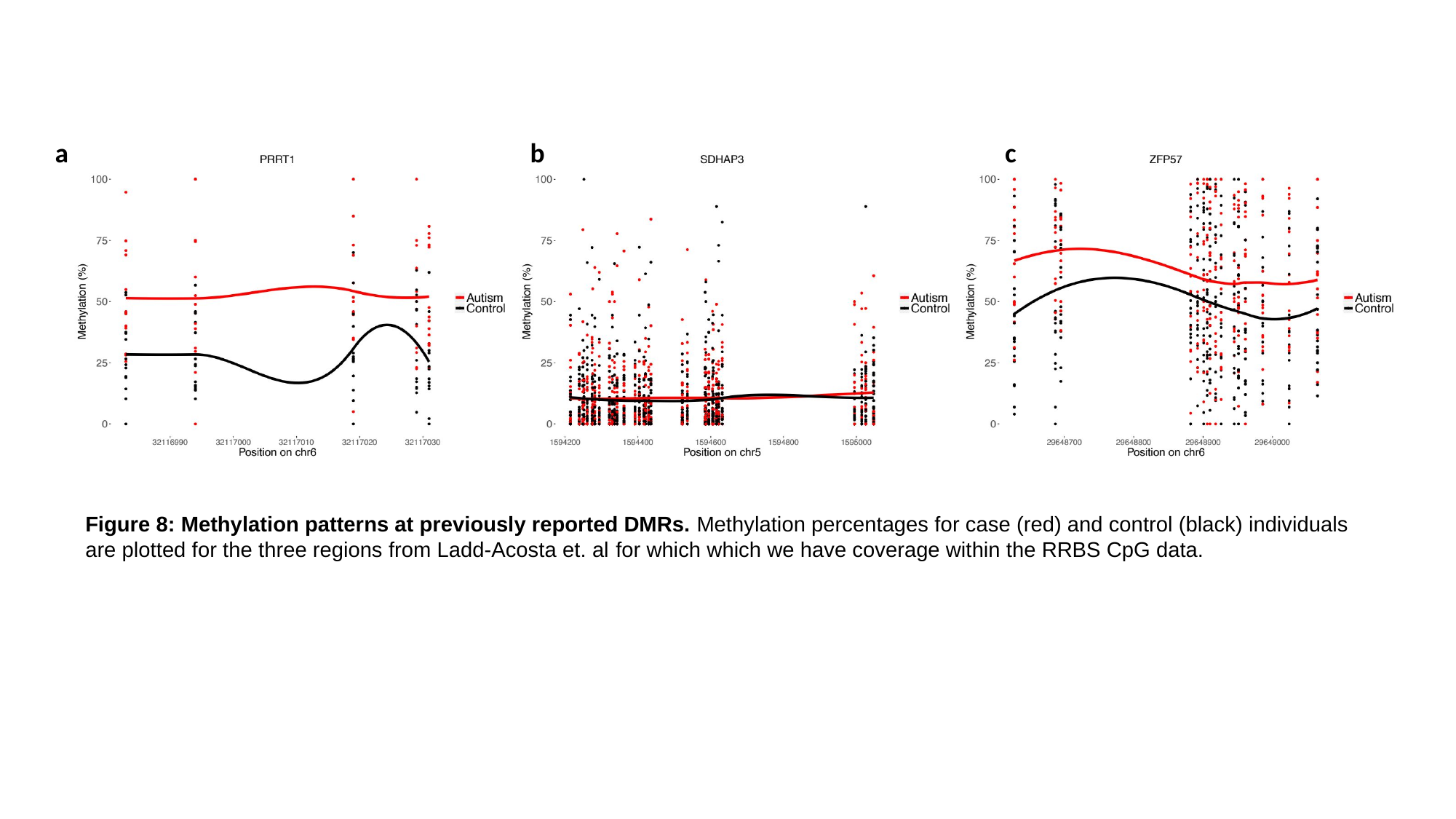

a
b
c
Figure 8: Methylation patterns at previously reported DMRs. Methylation percentages for case (red) and control (black) individuals are plotted for the three regions from Ladd-Acosta et. al for which which we have coverage within the RRBS CpG data.

## Slide 9
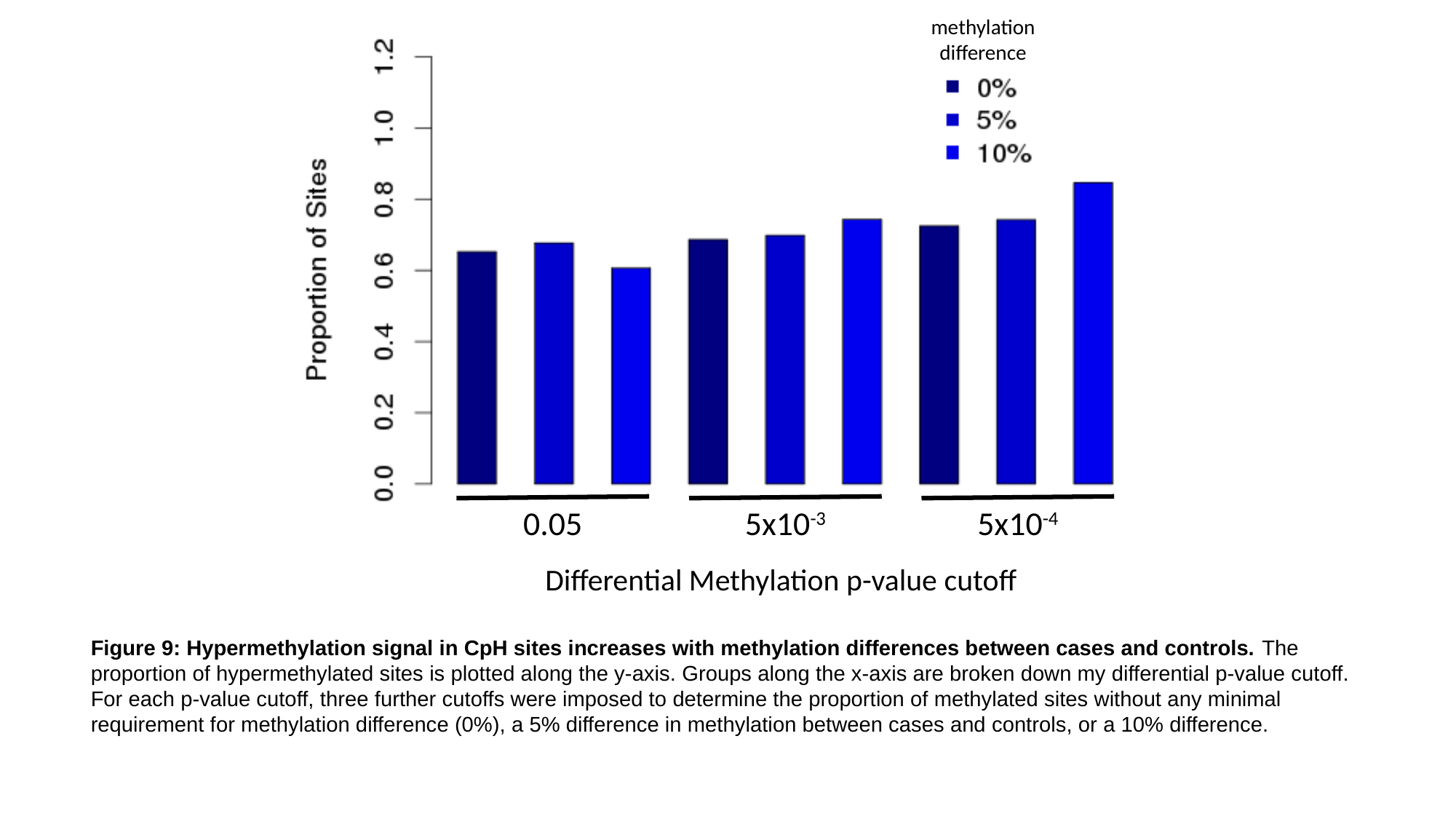

methylation difference
0.05
5x10^-3^
5x10^-4^
Differential Methylation p-value cutoff
Figure 9: Hypermethylation signal in CpH sites increases with methylation differences between cases and controls. The proportion of hypermethylated sites is plotted along the y-axis. Groups along the x-axis are broken down my differential p-value cutoff. For each p-value cutoff, three further cutoffs were imposed to determine the proportion of methylated sites without any minimal requirement for methylation difference (0%), a 5% difference in methylation between cases and controls, or a 10% difference.

## Slide 10
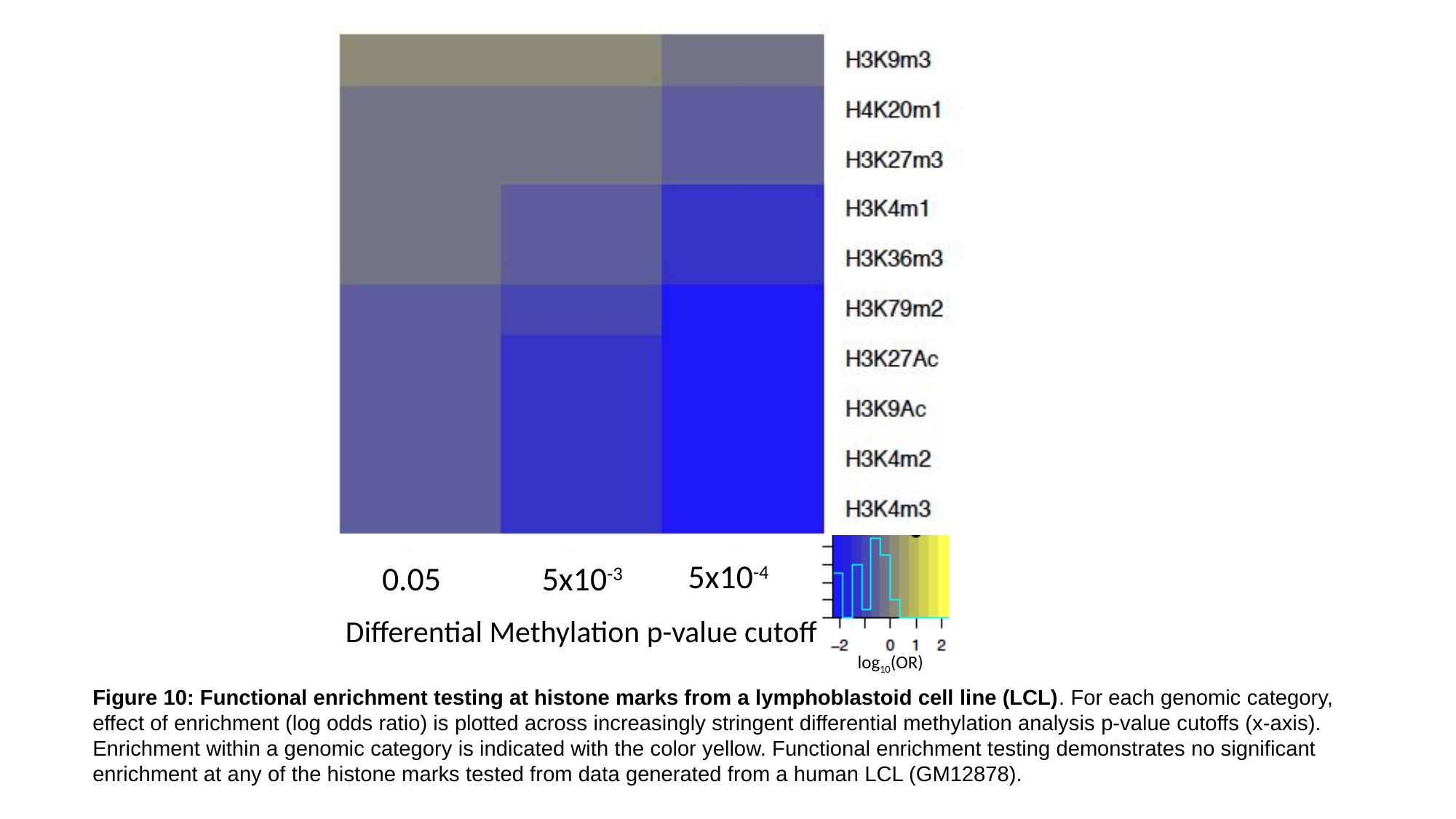

log10(OR)
5x10^-4^
5x10^-3^
0.05
Differential Methylation p-value cutoff
log10(OR)
Figure 10: Functional enrichment testing at histone marks from a lymphoblastoid cell line (LCL). For each genomic category, effect of enrichment (log odds ratio) is plotted across increasingly stringent differential methylation analysis p-value cutoffs (x-axis). Enrichment within a genomic category is indicated with the color yellow. Functional enrichment testing demonstrates no significant enrichment at any of the histone marks tested from data generated from a human LCL (GM12878).

## Slide 11
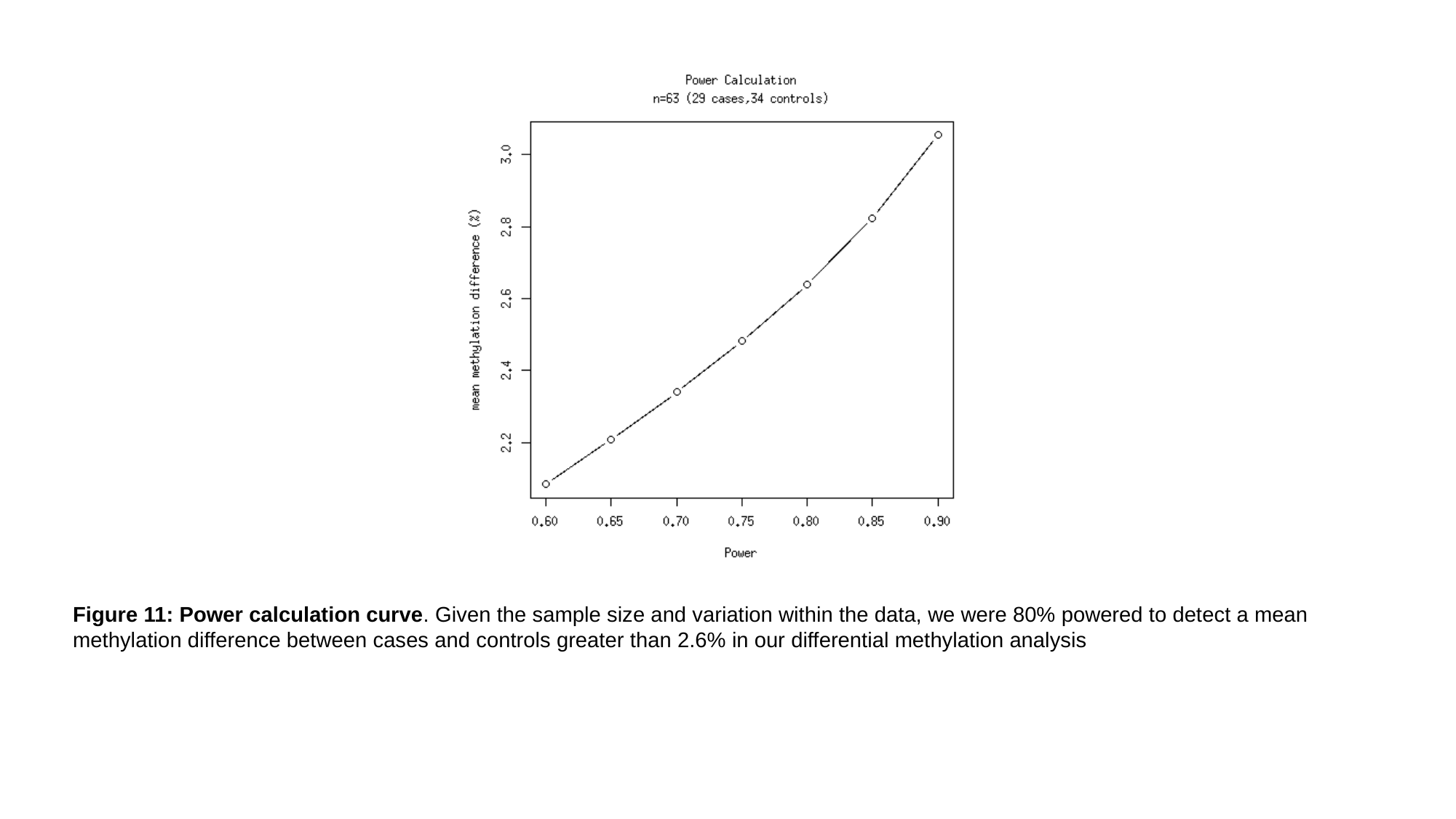

Figure 11: Power calculation curve. Given the sample size and variation within the data, we were 80% powered to detect a mean methylation difference between cases and controls greater than 2.6% in our differential methylation analysis
